# Effectiveness of the Air-Filled Technique to Reduce the Dead Space in Syringes and Needles during ChAdox1-n CoV Vaccine Administration

**DOI:** 10.3390/vaccines11040741

**Published:** 2023-03-27

**Authors:** Naphatthorn Prueksaanantakal, Anan Manomaipiboon, Patchara Phankavong, Warissara Jirawathin, Nontawat Benjakul, Jakravoot Maneerit, Uraporn Phumisantiphong, Thananda Trakarnvanich

**Affiliations:** 1 Kuakarun Faculty of Nursing, Navamindradhiraj University, Bangkok 10300, Thailand; 2 Faculty of Medicine, Vajira Hospital, Navamindradhiraj University, Bangkok 10300, Thailand

**Keywords:** air-filled technique, ChAdox1-n CoV vaccine, COVID-19, low dead space syringe

## Abstract

In the current study, we calculated the vaccine volume and amount of dead space in a syringe and needle during ChAdox1-n CoV vaccine administration using the air-filled technique. The aim is to reduce the dead space in syringes and needles in order to administer up to 12 doses per vial. The hypothetical situation uses a vial with a similar size as the ChAdox1-n CoV vial. We used distilled water (6.5 mL) to fill the same volume as five vials of ChAdox1-n CoV. When 0.48 mL of distilled water is drawn according to the number on the side of the barrel, an additional 0.10 mL of air can be used in the dead space of the distilled water in the syringe and needle for 60 doses, which can be divided into an average of 0.5 mL per dose. ChAdox1-n CoV was administered using a 1-mL syringe and 25G needle into 12 doses using this air-filled technique. The volume of the recipient vaccine will increase by 20% and save on the budget for low dead space syringes (LDS).

## 1. Introduction

Since December 2019, the SARS-CoV-2 virus epidemic has spread worldwide, leading to the declaration of a pandemic in March 2021 [1]. Several countries have endeavored to experiment and create vaccines to prevent and control the transmission of COVID-19 [2]. The main vaccine in Thailand is ChAdox1-n CoV, which has been approved by the Ministry of Public Health and the Food and Drug Administration. This vaccine was registered on 20 January 2021. However, the limited capacity and vaccination demand of every country has increased beyond supply, creating a shortage of vaccines in most countries, including Thailand.

ChAdox1-n CoV is used to enhance immunity among people ≥ 18 years of age to prevent symptoms of COVID-19 [3]. This vaccine is a monovalent vaccine consisting of adenovirus from chimpanzees (ChAdOx1) encrypted by the glycoprotein S of SARS-CoV-2 [4]. After injection, the glycoprotein S of SARS-CoV-2 strengthens the immune system and prepares them for later infection.

**Figure 1:**
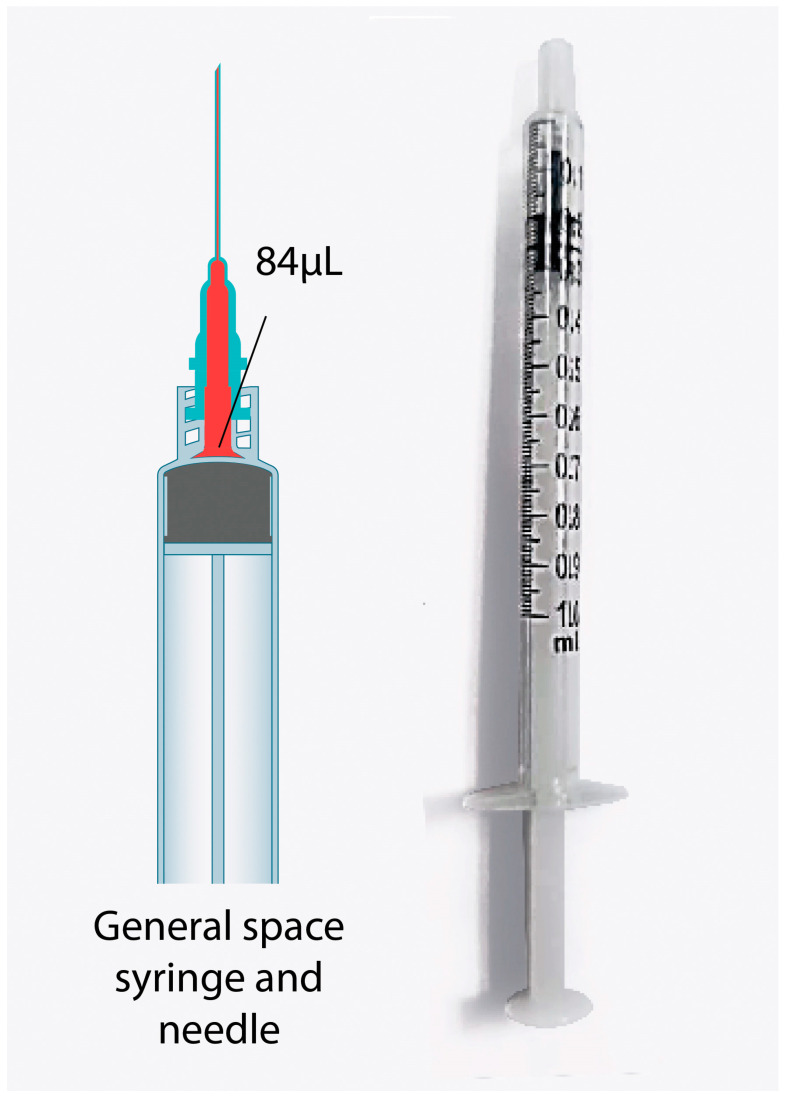
General space syringe and needle.

**Figure 2:**
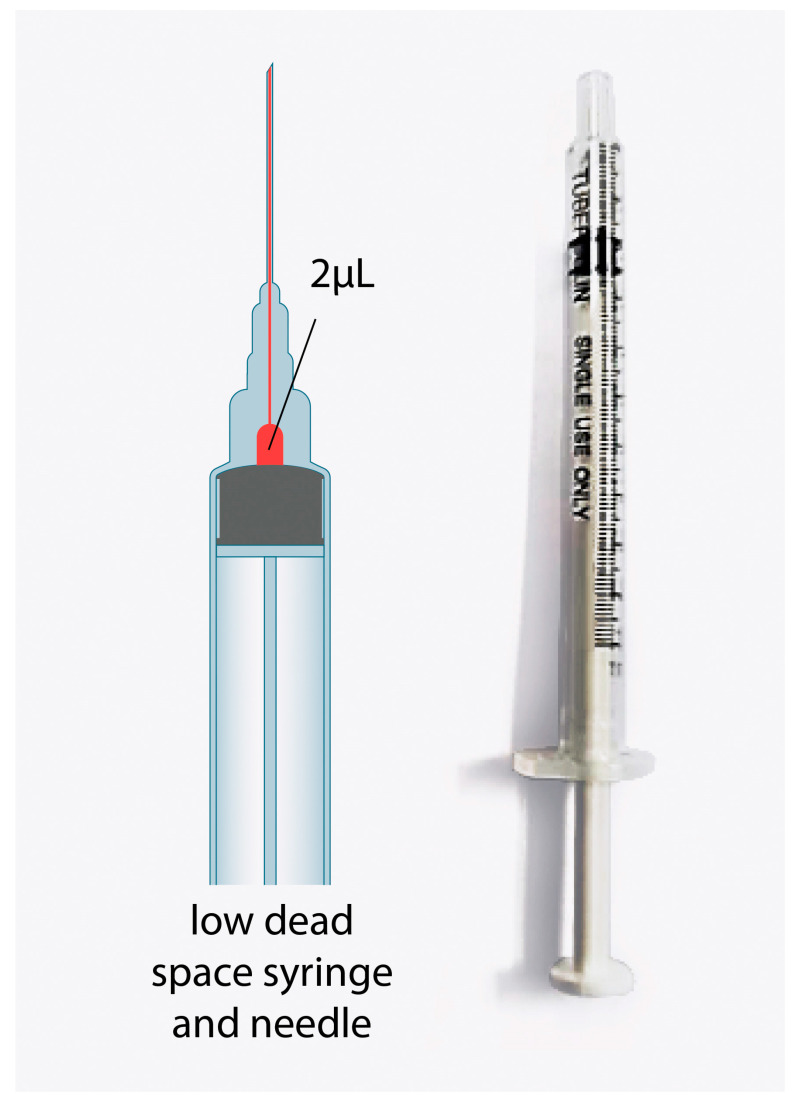
Low dead space syringe and needle.

A vial of ChAdox1-nCoV can be divided into ten doses (multiple doses) of 5 mL for ten people. The manufacturers generally overfill the vial to replace the dead space in the syringe and needle in the preparation and injection procedure for ten people. The amount of overfilled vaccine is the same in every brand for multiple dose vials, including Pfizer, which has sufficient vaccines for six people. Therefore, in many countries that have reserved COVID-19 vaccines, the multiple dose type is assigned to use low dead space (LDS) syringes and needles to reduce the wasted volume that remains in the tip [5]. However, the LDS and needle cost is 4 to 10 times more than a 1-mL syringe and 25G needle (1 to 1.5-inch length) (Figure 1 and Figure 2). Therefore, the latter syringes and needles are commonly used. Additionally, no manufacturers for LDS and needles are available in Thailand, and they need to be imported from overseas. Moreover, there is a worldwide shortage of vaccines. Therefore, a study about vaccine administration, including the preparation and injection procedure of ChAdox1-n CoV, to allow the vaccine to be divided into 12 doses per vial instead of 10, as limited by the manufacturer, is required. This procedure will help clinics suitably administer the vaccine in the most efficient manner, which will mitigate the supply issues and unavailability of LDS needles in many countries [6,7].

## 2. Materials and Methods

This was a single-center, prospective, observational study conducted at the Faculty of Medicine, Navamindradhiraj University, Bangkok, Thailand, and was approved by the ethics committee at our hospital, registration no. 096/2564. All procedures were performed after written informed consent from all participants. We conducted the experiment in two phases. The first phase was done with distilled water, and the second phase was the real vaccine.

The research procedure was as followed:

The test was first performed using distilled water to test the hypothetical situation.

A vial with a similar size as the ChAdox1-n CoV vial was used. Distilled water of the same volume as a single vial of ChAdox1-n CoV (6.5 mL) was filled.

A digital precision balance weighing scale was prepared to measure empty syringes and vaccines (in mg) up to four decimal places and calibrations from the National Institute of Metrology Thailand (NIMT), as described below. 

Step 1: Sixty sets of 1-mL syringes and 25G needles were weighed and recorded, with a length of 1 inch needles, and the weighted average was calculated.

Step 2: A small amount (0.45 mL) of water with 0.1 mL air was aspirated, resulting in <0.5 mL water being ejected from the syringe.

Step 3: A slightly larger amount (0.48 mL) of distilled water and an additional 0.10 mL of air were drawn into the syringe to reduce the dead space in the syringe and needle. The amount of the water appeared to be slightly more than 0.5 mL. Thereafter, the weights of 60 sets of syringes and needles were recorded, and the weighted average was calculated.

Step 4: A further 0.5 mL of distilled water from the barrel was discarded, the syringe and needle were weighed, the weights recorded, and the weighted average was calculated.

The experiment using the same procedures was repeated until 12 doses of distilled water were obtained per vial, for which the amount in every dose was ≥0.5 mL. 

**Figure 3:**
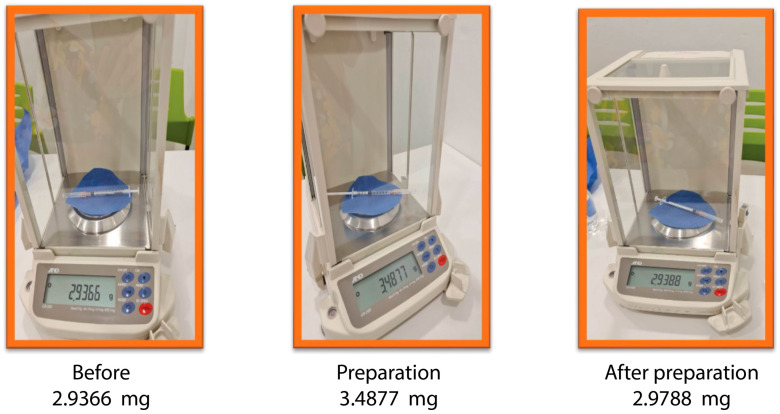
Comparison of vaccine weights by using the air-filled technique. Before: Weight of the empty syringe and needle. Preparation: Filled syringe with the vaccine. After preparation: Weights of the syringe and needle after injection.

The second stage involved using ChAdox1-n CoV in a real situation in the same way as in the hypothetical situation, as described below (Figure 3).

Step 1: Sixty sets of 1-mL syringes and 25G needles, with a length of a 1 inch needle, were weighed, the weights recorded, and the weighted average was calculated. Five vials of the vaccine were used. Since a vial can be divided into 12 doses, five vials would comprise 60 doses.

Step 2: The vaccine was prepared by stabbing the needle in the vaccine vial and drawing in 0.48 mL of vaccine and an additional 0.10 mL of air to reduce the dead space in the syringe and needle. The amount of the vaccine would appear to be equal to or slightly more than 0.5 mL. Thereafter, the 60 syringe and needle sets were weighed, the weights recorded, and the weighted average was calculated.

Step 3: The vaccine was injected into the muscle of the vaccine recipient following the vaccine administration instructions composed by the Nursing Department of the Faculty of Medicine, Vajira Hospital, Navamindradhiraj University. Thereafter, the 60 syringe and needle sets were weighed, the weights recorded, and the weighted average was calculated.

### 2.1. ChAdox1-n CoV Injection Technique

The vaccine injection using the same needles to draw the vaccine from the same vial could allow diseases to spread easily, such as hepatitis B, hepatitis C, and human immunodeficiency virus (HIV). Thus, the hospital and universal infectious control guidance precautions strictly adhered to the aseptic technique. The vaccine injection procedures are described below. 

1. Medical personnel must wear a surgical mask, glasses, and face shield and sanitize their hands with hand sanitizer and wear suitable gloves.

2. The medical personnel and recipients receiving the injection must put one of their upper arms on the table or on the surface, which is not higher than shoulder level, and not flex their shoulder and upper arm muscles during the injection. 

3. The syringe should be checked for whether it contains 0.5 mL of the vaccine as described by Astra Zeneca. The injection site should be cleaned with 70% alcohol and left to dry. 

4. Before injecting the vaccine, the syringe should be pointed 90 degrees to let the air flow to the top of the barrel. Flicking the finger lightly on the barrel helps the air bubble move to the top (the syringe should not be shaken). Next, the needle should be inserted through the subcutaneous layer to the muscle, ensuring that the tip of the needle is not in a blood vessel, and the vaccine injected by pushing the plunger to the end. The air on the top part of the syringe pushes the dead space in the syringe and needle into the muscle by a slight amount. Therefore, less than 1 mL of air might pass to the muscle, which does not cause any harm. If the medical personnel and recipients receiving an injection have a thick subcutaneous layer, a 1.5-length 25G needle should be used instead.

5. The needle should be pulled out from the vaccine recipient and removed to a needle clipping device without using the Luer lock. The syringe and all other equipment used should be discarded into the infectious waste disposal container.

6. Finally, a band-aid should be placed on the injection wound, and the vaccine recipient should be asked to wait in the waiting area to observe whether any symptoms occur after the injection. 

The researcher should check the specific gravity in the five vials of the ChAdox1-n CoV used in the experiment to calculate the volume that the patient has received.

### 2.2. Outcome Measurement 

The primary outcome was to evaluate the volume and weight of the vaccine after divided it into 12 doses per vial. The secondary outcomes were to test the accuracy of the 25G needle and normal syringe instead of the low dead space syringe. 

### 2.3. Statistical Analysis

Data were expressed as the mean ± SD in normal distribution data and median (IQR) in not normally distributed data. The data were analyzed using SPSS Program, Version 28 (Chulalongkorn University Bangkok Thailand) to calculate the weighted average of the syringe, needle, vaccine, volume, and standard variation.

## 3. Results

In the first experimental phase, we used 6.5 mL of the distilled water in one vial, which was the same size as the vaccine ChAdox1-n CoV vial. Then, we added 0.48 mL of distilled water and an additional 0.1 mL of air to replace the dead space of the distilled water in the syringe. The needle was then withdrawn. The distilled water was administered to 12 doses per vial. The weighted average in the syringe before injection was measured to be 0.5168 mL, the weighted average of dead space 0.0122 mL, and the volume of distilled water ejected from the syringe 0.5046 mL (Table 1).

The second stage of the ChAdox1-nCoV vaccine administration experiment was performed by using a normal syringe and needle to draw 0.48 mL of the vaccine, according to the number on the side of the barrel, and an additional 0.10 mL of air to reduce the dead space in the syringe and needle. We then used this procedure to prepare 0.5 mL or slightly more of the ChAdox1-n CoV vaccine using a 1-mL syringe and 25G needle to draw in 0.48 mL of the vaccine, according to the number on the side of the barrel, and an additional 0.10 mL of air to reduce the dead space in the syringe and needle. We, could therefore administer 12 doses of vaccine per vial. The weighted average of the vaccine before injecting was found to be 0.5048 mL, and the weighted average of the dead space 0.0263 mL. Thus, the volume of the vaccine that the recipient would receive was 0.5048 mL (the weight of the vaccine that the recipient would receive was =0.5199/1.030 (sp.gr. of the vaccine)) (Table 2).

## 4. Discussion

This experiment was aligned with research on the antibody level for SARS-CoV-2 after injection of the first dose of ChAdox1-n CoV-19 using 12 doses per vial [8]. We found that approximately 8.57 weeks after injecting 12 doses per vial, the level of immunity was higher at a positive value of 93.3% and higher among women than men. Additionally, immunity did not vary according to age, disease, body mass index, blood type, or blood pressure and corresponded with the advice from the Pfizer-BioNTech COVID-19 vaccine, which uses LDS to divide six doses of the vaccine per vial. We monitored both the human antibodies of the immunoglobulin class IgG against the SARS-CoV-2 S1/RBD spike protein and the neutralizing antibody (Nab) test. After injecting the vaccine into the recipient, we found ≤0.035 mL dead space in the syringe. From this experiment using the air-filled technique to reduce the dead space in the syringe and needle, 0.0263 mL of the vaccine remained. Thus, the vaccine recipient will receive 0.5048 mL volume of the vaccine using the 1-mL syringe and 25G needle instead of the LDS. This technique will reduce the expenses of importing LDS, which costs 2.78 Thai Baht (THB) per syringe compared to 1.65 THB for a 1 mL syringe and 0.43 THB for a 25G needle. This technique of vaccine administration increases the amount of available vaccine doses by 20% and provides a cheaper alternative to the LDS syringe. This will help the Nursing Department of the Faculty of Medicine, Vajira Hospital, to create guidelines and strategies to administer the vaccine using the air-filled technique to reduce the dead space in the syringe and needle, as well as to publish an interdisciplinary method in vaccine administration for all vaccine recipients. These data have the potential to optimize limited vaccine resources available during this global health problem emergency, as additional doses may increase the number of people vaccinated. Moreover, these dead volume data can be extrapolated to other multi-dose COVID-19 vaccines and may allow the use of additional doses by taking advantage of overfilling.

The strength of this study is that we created a novel technique that can enhance reproducibility and achieved good immunogenicity. The limitations are the small sample size and the inter-variability between each personnel. This can be overcome by repetitive training for good reproducibility. 

## 5. Conclusions

ChAdox1-n CoV vaccine administration using a 1-mL syringe and 25G needle to draw in 0.48 mL of vaccine, according to the number on the side of the barrel, and an additional 0.10 mL of air to reduce the dead space in a syringe and needle to achieve an average volume of 0.5 mL (or slightly more) allowed the vaccine to be administered at 12 doses per vial. The air-filled and vertical injection techniques included flicking the finger lightly on the barrel to let the air flow to the top of the barrel. Then, the syringe was pointed at 90 degrees to pierce the muscle before pushing the plunger to the end. The air on the top pushed the dead space at the tip of the syringe and needle. We found that 0.0263 mL of dead space remained. Vaccinations with 12 doses per vial elicited a good humoral response against SARS-CoV-2. Nearly all of the participants achieved acceptable antibody levels, while more than 50% exhibited high levels of neutralizing antibodies. The side effects were few. We therefore recommend this air-filled technique to optimize vaccines and achieve their maximal benefit.

## Figures and Tables

**Table 1:** First stage using distilled water in the hypothetical situation.

Vial No.	Syringe Needle Weight		Syringe Needle Weight after Drawing the Distilled Water		Weight of Distilled Water in Syringe before Injection		Weight of Distilled Water in Syringe after Injection		Weight of the Dead Space of Distilled Water in Syringe		Volume of Distilled Water the Patient Received	
	Mean	SD	Mean	SD	Mean	SD	Mean	SD	Mean	SD	Mean	SD
Vial 1	2.9408	0.0153	3.4560	0.0136	0.5151	0.0071	2.9549	0.0111	0.0141	0.0084	0.5011	0.0084
Vial 2	2.9348	0.0175	3.4542	0.5194	0.5194	0.0074	2.9510	0.0172	0.0161	0.0024	0.5033	0.0078
Vial 3	2.9330	0.0091	3.4516	0.0180	0.5186	0.0137	2.9428	0.0102	0.0098	0.0073	0.5089	0.0128
Vial 4	2.9351	0.0083	3.4501	0.0155	0.5149	0.0137	2.9457	0.0115	0.0105	0.0094	0.5044	0.0122
Vial 5	2.9359	0.0081	3.4516	0.0130	0.5157	0.0118	2.9462	0.0083	0.0103	0.0080	0.5054	0.0099
Average	2.9359	0.0122	3.4527	0.0148	0.5168	0.0109	2.9481	0.0124	0.0122	0.0077	0.5046	0.0104

**Table 2:** The second stage using ChAdox1-n CoV in the real situation.

Vial No.	Syringe Needle Weight		Syringe Needle Weight after Drawing the Vaccine		Weight of Vaccine in Syringe before Injection		Weight of Vaccine in Syringe after Injection		Weight of the Dead Space of Vaccine in Syringe		Weight of Vaccine Injected out of the Syringe		Volume of Vaccine the Patient Received	
	Mean	SD	Mean	SD	Mean	SD	Mean	SD	Mean	SD	Mean	SD	Mean	SD
Vial 1	2.9326	0.0133	3.4804	0.0119	0.5478	0.0144	2.9566	0.0101	0.0240	0.0069	0.5238	0.0081	0.5086	0.0079
Vial 2	2.9316	0.0310	3.4833	0.0184	0.5517	0.0213	2.9737	0.0219	0.0421	0.0244	0.5095	0.0134	0.4947	0.0130
Vial 3	2.9436	0.0227	3.4861	0.0220	0.5425	0.0043	2.9654	0.0234	0.0218	0.0043	0.5207	0.0045	0.5055	0.0044
Vial 4	2.9345	0.0112	3.4759	0.0124	0.5414	0.0048	2.9540	0.0114	0.0195	0.0055	0.5219	0.0050	0.5067	0.0048
Vial 5	2.9337	0.0136	3.4819	0.0130	0.5482	0.0132	2.9576	0.0101	0.0239	0.0066	0.5243	0.0080	0.5090	0.0078
Average	2.9353	0.0205	3.4816	0.0165	0.5462	0.0137	2.9616	0.0182	0.0263	0.0148	0.5199	0.0100	0.5048	0.0097

## Data Availability

All data used in the preparation of the manuscript are related to the previously published article and can be found in the Mendeley Data repository: “ChAdOx1nCoV-19 vaccination”, Mendeley Data, V1, doi: 10.17632/yjhssw33cw.1.
